# Insights into water insecurity in Indigenous communities in Canada: assessing microbial risks and innovative solutions, a multifaceted review

**DOI:** 10.7717/peerj.18277

**Published:** 2024-10-18

**Authors:** Jocelyn I. Zambrano-Alvarado, Miguel I. Uyaguari-Diaz

**Affiliations:** Department of Microbiology, Faculty of Science, University of Manitoba, Winnipeg, Manitoba, Canada

**Keywords:** Indigenous communities, Water insecurity, Water, Canada, Microbiology, Drinking water systems, Indigenous reserves, Bacteria, Virus, Metagenomics

## Abstract

Canada is considered a freshwater-rich country, despite this, several Indigenous reserves struggle with household water insecurity. In fact, some of these communities have lacked access to safe water for almost 30 years. Water quality in Canadian Indigenous reserves is influenced by several factors including source water quality, drinking water treatments applied, water distribution systems, and water storage tanks when piped water is unavailable. The objective of this multifaceted review is to spot the challenges and consequences of inadequate drinking water systems (DWS) and the available technical and microbiological alternatives to address water sanitation coverage in Indigenous reserves of Canada, North America (also known as Turtle Island). A comprehensive literature review was conducted using national web portals from both federal and provincial governments, as well as academic databases to identify the following topics: The status of water insecurity in Indigenous communities across Canada; Microbiological, chemical, and natural causes contributing to water insecurity; Limitations of applying urban-style drinking water systems in Indigenous reserves in Canada and the management of DWS for Indigenous communities in other high-income countries; and the importance of determining the microbiome inhabiting drinking water systems along with the cutting-edge technology available for its analysis. A total of 169 scientific articles matched the inclusion criteria. The major themes discussed include: The status of water insecurity and water advisories in Canada; the risks of pathogenic microorganisms (*i.e.*, *Escherichia coli and* total coliforms) and other chemicals (*i.e.*, disinfection by-products) found in water storage tanks; the most common technologies available for water treatment including coagulation, high- and low-pressure membrane filtration procedures, ozone, ion exchange, and biological ion exchange and their limitations when applying them in remote Indigenous communities. Furthermore, we reviewed the benefits and drawbacks that high throughput tools such as metagenomics (the study of genomes of microbial communities), culturomics (a high-efficiency culture approach), and microfluidics devices (microminiaturized instruments) and what they could represent for water monitoring in Indigenous reserves. This multifaceted review demonstrates that water insecurity in Canada is a reflection of the institutional structures of marginalization that persist in the country and other parts of Turtle Island. DWS on Indigenous reserves are in urgent need of upgrades. Source water protection, and drinking water monitoring plus a comprehensive design of culturally adapted, and sustainable water services are required. Collaborative efforts between First Nations authorities and federal, provincial, and territorial governments are imperative to ensure equitable access to safe drinking water in Indigenous reserves.

## Introduction

Canada contains the fourth largest freshwater reserve in the world, only surpassed by Brazil, Russia, and the United States of America (Natural Resources Canada, 2017; Desforges et al., 2022). Although the vast majority of Canadians have access to piped potable water supply (as expected in a freshwater-rich country), numerous Indigenous reserves across Turtle Island struggle with household water insecurity (Basdeo & Bharadwaj, 2013; Brown et al., 2016; Waldner et al., 2017; Indigenous Services Canada, 2020; Wu et al., 2020; Lucier et al., 2020; Ratelle et al., 2022). Household water insecurity can be defined as the absence of safe, reliable, and affordable water (Meehan et al., 2020), and systemic disparities in a high-income country such as Canada make this scenario plausible. Despite the alleged efforts from the federal government to eliminate all long-term drinking water advisories in Canada by March 2021 (Office of the Auditor General of Canada, 2021), three years after the deadline, the issue still persists. Water advisories are issued by local authorities to communities to warn about water consumption or completely restrict its usage. It is estimated that in Canada there are more than 100 short-term drinking water advisories at any given time (Indigenous Services Canada, 2024a). As of September 2024, there are more than 30 long-term drinking water advisories remaining in Indigenous reserves (Indigenous Services Canada, 2024a). Water insecurity in Indigenous reserves is a multidimensional matter that started with the colonial past and continued with the deterioration of water, the environment, and other complex socio-economic structures existing in Canada (Sarkar, Hanrahan & Hudson, 2015). In this multifaceted review, we first explore the water advisories remaining in Indigenous reserves in the country. Moreover, we analyze additional factors that contribute to water insecurity in First Nations (FN) communities such as cisterns, water storage facilities, pathogenic bacteria, and other genetic elements such as antibiotic-resistance genes that have been reported in water locations of some Indigenous reserves. We also review the negative effects of high levels of natural organic matter (NOM) in drinking water systems (DWS). Moreover, we discuss some methods available for drinking water treatment and NOM removal such as coagulation, high- and low-pressure membrane filtration, ozone, Biological activated carbon filters, Ion exchange, and Biological Ion exchange. Additionally, we address the challenges of applying each of these methods in Indigenous reserves. Furthermore, we briefly explore the drinking water facilities available in Indigenous communities of other high-income countries such as the United States of America (USA), Australia, and some Nordic regions such as Greenland (Denmark), Finland, Norway, and Sweden. Finally, we review high throughput tools such as metagenomics, culturomics, and microfluidics devices that can provide important information regarding microbial communities and microbial pollutants present in source water and DWS in Indigenous reserves. This investigation synthesizes existing literature and Canadian public records concerning the prolonged state of water insecurity in Indigenous reserves to promote comprehension of the microbiological and chemical risks that this represents for public health. Several factors influence water quality including the source water quality, the drinking water treatment applied, the water distribution system, and water storage tanks. In the past few years, notable investment from the federal government of Canada has led to solving 145 long-term drinking water advisories in Indigenous reserves (Employment and Social Development Canada, 2024). However, some communities haven’t had access to safe water for almost 30 years (Indigenous Services Canada, 2024b). This review aims to spot the challenges and consequences of inadequate DWS and the available technical and microbiological alternatives to address water sanitation coverage in Indigenous reserves of Turtle Island. In a high-income country like Canada, water insecurity tends to get overshadowed with more than 98% of the population having water sanitation coverage (Balasooriya, Rajapakse & Gallage, 2023). With this study, we want to raise awareness for the minority facing water insecurity advocating for the prioritization of resources to ensure they have access to an internationally recognized human right which is access to safe water and sanitation (Office of the United Nations High Commissioner (OHCHR), 2015). While we restricted the scope of this review to technical and microbiological issues, we acknowledge that water insecurity is the result of socio-cultural, systemic disparities, historic and institutionalized marginalization of Indigenous people, and an ineffective and segregated system of water governance that applies to FN reserves (Meehan et al., 2020).

In this multifaceted review, we discuss technical innovations and advanced molecular approaches that could be instrumental in addressing and ultimately overcoming long-term drinking water advisories. Presently, water quality monitoring practices assess fecal contamination by detecting indicator bacteria, including coliforms like *Escherichia coli* (*E. coli*) and enterococci. These indicator microorganisms were established more than a century ago, and they have been proven ineffective in assessing water for other microorganisms such as viral, or protozoan pathogens (Ramírez-Castillo et al., 2015). Advanced molecular tests reviewed in this article can complement the assessment of other pathogens and indicator microorganisms present in drinking water to help ensure safe water in Indigenous reserves. This article is one of the few that reviews both technical and advanced molecular tools that could be applied in remote Indigenous communities and is useful for researchers involved in the fields of environmental sciences, microbiology, water treatment engineering, and Indigenous studies.

## Methodology

### Database search and literature screening

Federal and provincial government portals were used to review the status of water advisories in Indigenous communities of Canada as well as the recommended guidelines for NOM and drinking water in the country. The federal government web pages included in this review are “Indigenous Services Canada”, “Environment and Climate Change Canada”, “Health Canada”, “Statistics Canada”, “Employment and Social Development Canada”, “Natural Resources Canada”, “Canada’s Chief Public Health Officer”, “Office of the Auditor General of Canada”, “Standards Council of Canada”, “Province of Manitoba” and “Government of Ontario”. Three official international government portals were reviewed to examine the drinking water guidelines of the countries mentioned in this study including “National Health and Medical Research Council” for Australian drinking water Guidelines, “U.S. Environmental Protection Agency” for the US drinking water regulations and the “European Commission” for the Eropean Union Drinking Water Directive. Furthermore, PubMed and Google Scholar databases were used to identify relevant literature. To ensure a comprehensive exploration of the topics, a web search was conducted including the following keywords: “Indigenous communities” or “Indigenous reserves” or “First Nations” combined with “Canada”. The terms “water insecurity” and “natural organic matter” and “drinking water” and “Drinking water advisories” or “boil water advisory” or “drinking water systems” or “drinking water treatments” or “drinking water facilities” were also used. Finally, to include the microbiological approaches reviewed the terms: “Microbiology” or “Uncultivable bacteria” or “Metagenomic” or “DNA sequencing” were used. Portions of this text were previously published as part of a preprint (https://doi.org/10.31219/osf.io/w5hxy).

## Results

Peer-reviewed publications, theses, and government portals addressing the following topics were retained for the final report: Water insecurity in Indigenous communities in Canada, DWS in Indigenous communities, Drinking water treatments, NOM, water storage tanks in Indigenous communities in Canada, microorganisms, metagenomics for water monitoring, culturomics, and microfluidics. A total of 169 scientific articles matched the topics explored. Eighteen government portals (15 national, three international) were included in the final review. The four major dimensions discussed include:

• Water advisories.
• Microbiological, chemical, and natural causes contributing to water insecurity.
• Limitations of applying urban-style drinking water systems in Indigenous reserves in Canada and the management of DWS for Indigenous communities in other high-income countries.
• The importance of determining the microbiome inhabiting drinking water systems and the cutting-edge technology available for its analysis.

### Synthesis of results and discussion

### Water advisories in Canada

**Figure 1:**
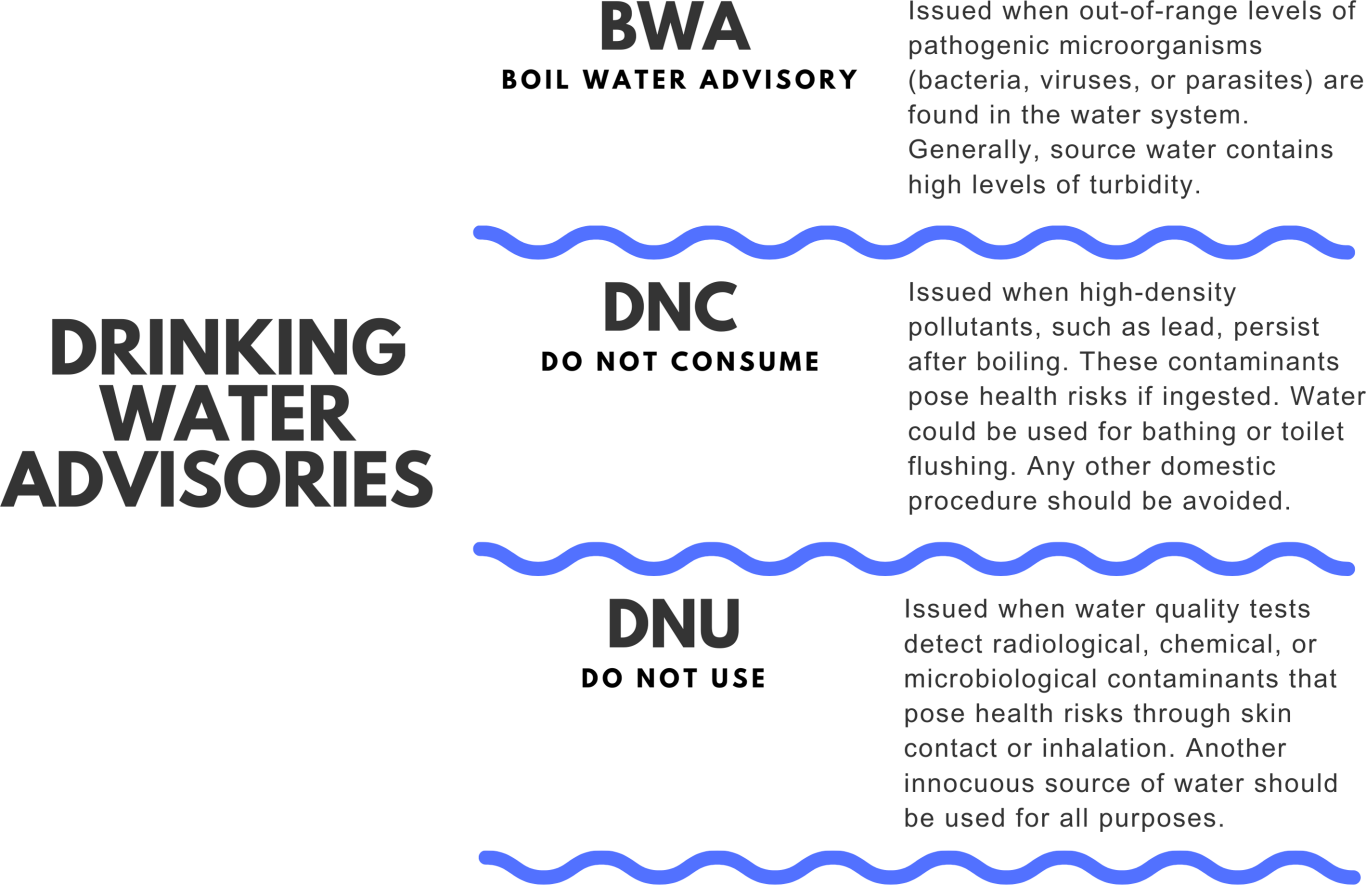
Drinking water advisories (DWA). Types of drinking water advisories (DWA) issued in Canada. Boil water advisories (BWA), do not consume (DNC), and do not use (DNU). BWA represents the majority of DWA issued in Canada.

In Canada, the forced relocation of Indigenous people caused the DWS in Indigenous communities to be placed in remote locations (Statistics Canada, 2022). Therefore these DWS are susceptible to irregular connectivity, a limited number of qualified personnel on site, and generally depend on sporadic water operation and maintenance (Basdeo & Bharadwaj, 2013; Islam & Yuan, 2018; Lucier et al., 2020; Zambrano-Alvarado & Uyaguari, 2024). Moreover, several DWS on reserves have been reported to lack modern infrastructure and are in urgent need of significant upgrades (Zimmermann et al., 2020; Lucier et al., 2020). Additionally, in Indigenous reserves, water delivery, and maintenance responsibilities are shared between the Federal Government, specifically Indigenous Services Canada and Health Canada with FN community leadership groups (Boyd, 2011; Bradford et al., 2016; Lucier et al., 2020). This shared administration and fragmented responsibilities has led to divergences in drinking water regulations in Indigenous reserves (Boyd, 2011; Bradford et al., 2016). In addition, Indigenous communities’ dependence on federal funding to improve DWS, along with the slow and often delayed response from the government within a segregated and flawed system of governance, also contributes to Indigenous reserves experiencing water insecurity (Bradford et al., 2016; Ayotunde, 2017; Kiedrowski, Jones & Ruddell, 2017; Lucier et al., 2020). Water insecurity on reserves represents a health threat and could be one of the causes of FN populations having the lowest projected life expectancies across Canada (Statistics Canada, 2017). In the country, most water-related activities are the responsibility of the corresponding authority of that province or territory, and the obligations regarding monitoring water contaminants differ vastly from province to province (Hill et al., 2006; Environment and Climate Change Canada, 2015; Environment and Climate Change Canada, 2017). In some provinces of the country, when potential hazards are identified in the water sources of public water systems (*i.e.,* *E. coli*, Cyanobacteria blooms, disinfection by-products or DBP), local environmental authorities may issue alerts to the public known as “water advisories” to warn about the water consumption or ban its use completely (Environment and Climate Change Canada, 2020). The length of the advisory could be less than a year (short-term advisory) or longer (long-term advisory) (Environment and Climate Change Canada, 2020; Province of Manitoba, 2021; Indigenous Services Canada, 2024a). Depending on the results of water quality, the nature of the water issue encountered, and a risk evaluation approach to the conditions of the place of the DWS, different warnings may be issued by the responsible environmental public health officer (Environment and Climate Change Canada, 2020; Government of Canada, 2021; Environment and Climate Change Manitoba, 2024). Boiling water advisories (BWA), Do not consume (DNC), and do not use (DNU) are the types of advisories recommended by Environment and Climate Change Canada (Environment and Climate Change Canada, 2020; Environment and Climate Change Canada, 2022). BWA, the most common type of advisories, are most of the times precautionary and generally issued when poor water disinfection, deficient filtration, pressure loss in the distribution system, or inadequate maintenance of the equipment used to treat water is recognized (Health Canada, 2015). Similarly, DNC (also referred to as “do not drink”) and DNU advisories are recommended during emergencies (Health Canada, 2009). For example, catastrophic events, chemical spills, or other pollutants that affect human health after short-term exposure, unexpected changes in the physical characteristics of water, or invasion of undetermined contaminants through cross-connection problems (Health Canada, 2009). When the contaminant present in water can alter human health only through ingestion, a DNC advisory is issued. (Health Canada, 2015). On the other hand, DNU recommendations are communicated when the existing pollutant has an effect through dermal and/or inhalation contact (Fig. 1) (Health Canada, 2009; Health Canada, 2015). Yet, every province and territory uses its own terminology to issue its water quality recommendations and some regions (such as Ontario, Alberta, and some provinces of the Arctic) do not report water advisories in minor drinking water systems (Ministry of Health Ontario, 2011; Government of Canada, 2021; Water today Canada, 2021). Since 2015, there have been more than 100 long-term water advisories lifted on reserves around the country (Government of Canada, 2021; Indigenous Services Canada, 2024a; Indigenous Services Canada, 2024b).

**Figure 2:**
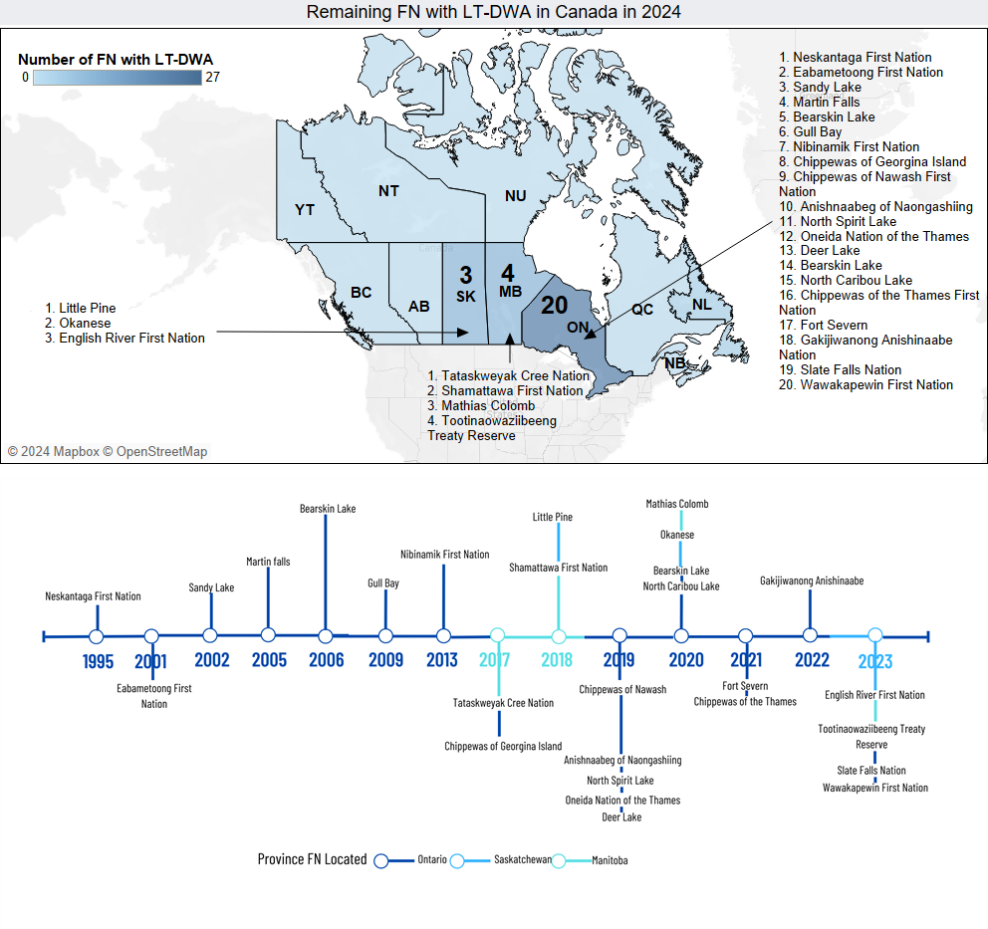
Remaining First Nations (FN) with long-term drinking water advisories (LTDWA) in Canada in 2024. (Top) Map based on the number of unresolved, long-term drinking water advisories (LT-DWA) issued around Canada: Saskatchewan = 3, Manitoba = 4, Ontario = 20. Note: provinces and territories colored with light blue have no LT-DWA to address or the information is unavailable. (Bottom) Timeline displaying the start year of the current unresolved LT-DWA across the country. Last updated: September 2024.

Nevertheless, as of September 2024, there are approximately 27 FN with long-term drinking water advisories on public systems on reserves (Indigenous Services Canada, 2024a; Indigenous Services Canada, 2024b). Astonishingly some of them have been dealing with advisories for almost 30 years. (Fig. 2) (Indigenous Services Canada, 2024b). Regarding short-term BWA, official sources only report the ones located south of the 60 parallel (Indigenous Services Canada, 2023). However, when contemplating all provinces and territories of Canada the estimated number of BWA advisories to be solved might exceed 1,000 cases. (Canada’s Chief Public Health Officer, 2013; Government of Canada, 2021; Water today Canada, 2021; Indigenous Services Canada, 2024b).

### Microbiological and chemical risks of water storage containers in Indigenous reserves

In FN communities in Canada, the DWS infrastructure has been reported to be either utterly absent, inappropriate, obsolete, or low quality (Lebel, 2008; Indigenous Services Canada, 2024a; Indigenous Services Canada, 2024b). The usage of wells, and trucked water to water storage facilities in households such as cisterns when a potable source of water is not found contributes to water insecurity. (Neegan Burside, 2011; Murdock et al., 2024). The construction materials accepted by the Canadian Standards Association include steel, stainless steel, concrete, reinforced concrete, fiberglass, and polymers (*i.e.,* polyethylene) (Standards Council of Canada, 2020). The materials and components used in water storage tanks are critical to maintaining water quality (World Health Organization, 2014). One example of this is the use of corrosive materials that harbor the development of iron and iron/manganese-oxidizing bacteria such as *Gallionella spp*. and members of the Siderocapsa genus (Kobrin & Lamb, 1997; Slavik et al., 2020). Generally, iron-oxidizing (*i.e.,* *Gallionella spp*.) and sulfur-reducing bacteria such as *Desulfovibrio spp*. are responsible for microbial-induced corrosion and biofilm development on unveiled metal surfaces(Annuk & Moran, 2010). Although biofilm development in these types of water storage units might represent a class of “blockade” to corrosion, it can also increase the risk of pathogen development that can potentially affect human health (Slavik et al., 2020). The complex heterogenous microorganisms present in biofilms can communicate through chemical interactions (known as “quorum sensing”) to facilitate multicellular activities, exchange nutrients, and transfer hereditary material (Wirthlin, Marshall & Rowland, 2003; Flemming, 2008). In terms of biofilm growth, particularly, iron water storage tanks have been found to harbor higher total bacteria counts than their counterparts made of plastic (Rilstone et al., 2021). Nevertheless, plastic cisterns have been associated with the presence of unacceptable levels of metals such as lead, aluminum, copper, among others, and even carcinogenic compounds (*i.e.,* benzene) in potable water (Moura et al., 2019). Moreover, high temperatures, changes in pH, and the presence of chloramines can influence the degradation of the polymeric matrix of these types of cisterns and favor the transference of the toxic compounds to stored water (Moura et al., 2019). Equally important is to ensure proper closure of the cisterns and avoid leakages (Artiola, Rock & Hix, 2012; Slavik et al., 2020). Algae, fungi, protozoa, bacteria, and viruses for example, can enter from windblown dust, debris, and rainwater if the water storage tank is not properly sealed (Benskin et al., 2009; Artiola, Rock & Hix, 2012). Additionally, the presence of leakages in the storage unit could allow the introduction of bird feces which are known to carry harmful bacteria such as *Salmonella* spp. and *Campylobacter* spp. (Benskin et al., 2009; Artiola, Rock & Hix, 2012; Slavik et al., 2020). The recommendations for these water storage tanks state that testing the water at least once per year is advisable to validate the presence/absence of microbiological pathogens (*i.e.,* total coliforms and *E. coli*) (Health Canada, 2009; Green Communities Canada, 2017). Nonetheless, some studies have confirmed that the water quality in these water storage tanks is below the stipulated standards (Fernando et al., 2016; Farenhorst et al., 2017; Murdock et al., 2024). The guidelines for Canadian drinking water quality state that the maximum contaminant level (MCL) for *E. coli* and total coliforms is 0 CFU/100 mL of water (Health Canada, 2013a; Health Canada, 2013b; Conservation and Water Stewardship Manitoba, 2014). However, *E. coli* counts higher than 60,000 CFU/100 mL were detected in drinking water distribution systems in a fly-in FN community in the Island Lake region in the province of Manitoba (Farenhorst et al., 2017).

**Figure 3:**
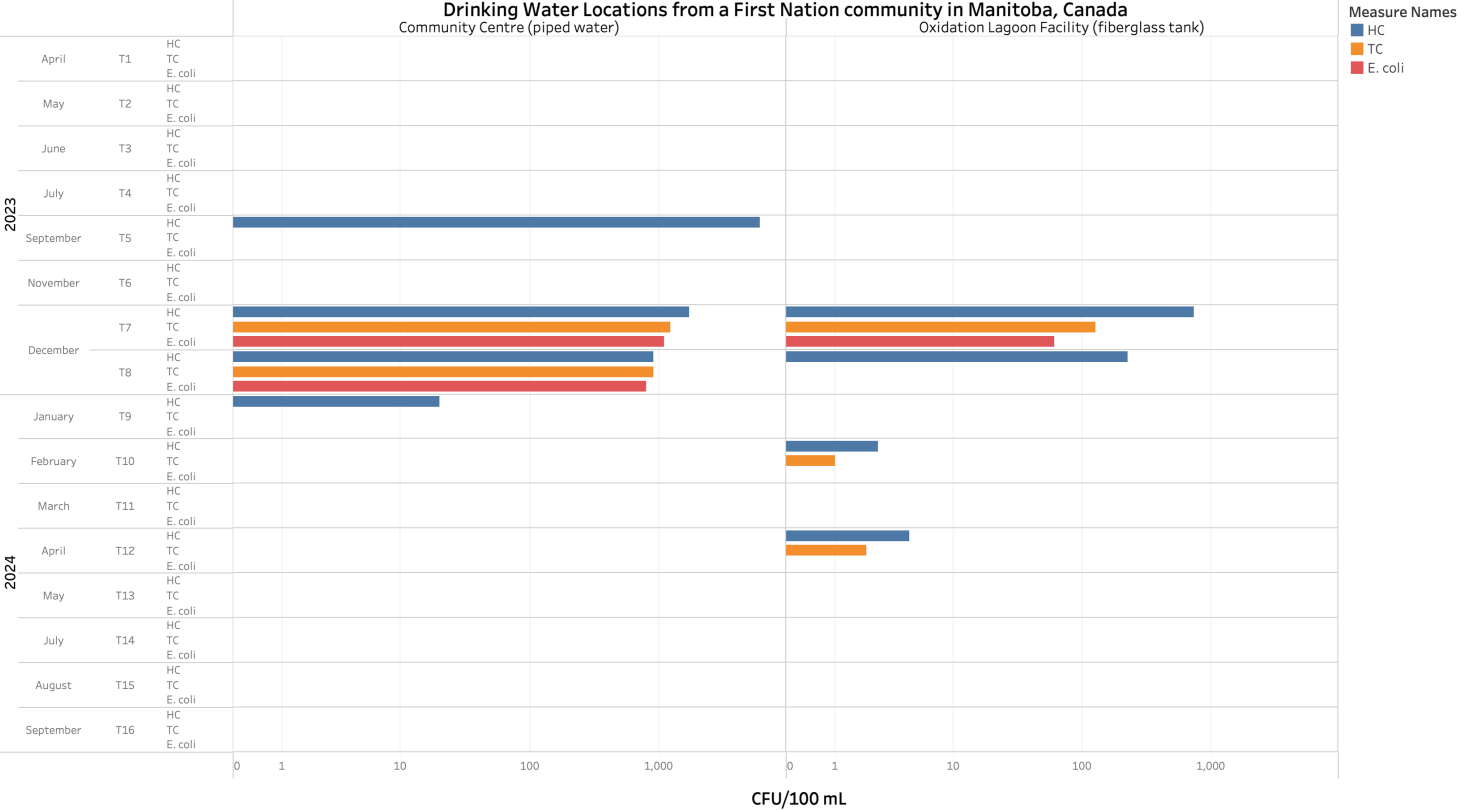
Microbiological counts observed per 100 ml of drinking water samples from a First Nation community in Manitoba, Canada. *E. coli*, total coliforms (TC), and heterotrophic plate counts (HC) from tap water collected from a community centre (piped water) and oxidation lagoon facility (fiberglass tank) in a First Nation community of Manitoba. Duplicate counts were assessed during 16 different time periods during April 2023 and September 2024. Unacceptable levels of *E. coli* appeared in both locations during December 2023. Exceeding counts for TC were also observed in both locations during December 2023, and in the fiberglass tank specifically during February and April 2024.

Likewise, unacceptable levels of *E. coli* (>1,000 CFU/100 mL, >900 CFU/100 mL, and >50 CFU/100 mL) were found in piped water and in a fiberglass tank used to store water for consumption in a FN community located in Manitoba (M. Miniruzzaman & M. Uyaguari-Diaz, 2024, unpublished data). Moreover, in the same community, total coliform counts exceeded acceptable detection limits (>1,000 CFU/100 mL, >900 CFU/100 mL, >50 CFU/100 mL, >0 CFU/100 mL and >1 CFU/100 mL) in two different locations in three out of 16 sampling events conducted from April 2023 to September 2024 (Fig. 3). Additionally, high heterotrophic counts (>1,000 CFU/100 mL and >500 CFU/100 mL) were reported in both the piped water and the fiberglass tank within the same community (M. Moniruzzaman & M. Uyaguari-Diaz, 2024, unpublished data). Even though heterotrophic bacteria do not represent a direct threat to public health and the counts obtained did not exceed the maximum acceptable concentrations (500 CFU/ mL), these high counts can interfere with *E. coli* and total coliforms recovery methods (Health Canada, 2013a; Health Canada, 2013b). Additionally, lower heterotrophic counts have been associated with better maintenance of the water facilities (Chowdhury, 2012; Health Canada, 2013b). Furthermore, the presence of antibiotic resistance genes (ARGs) such as *ampC* (*β*-lactam resistance), *mecA* (methicillin resistance), and *sul1* (sulfonamides resistance) in both, source, and drinking water in reserves of FN Communities have been reported (Fernando et al., 2016; Farenhorst et al., 2017; Mi et al., 2019). Moreover, the isolation of pathogenic bacteria such as *Legionella pneumophila* (MacMartin et al., 2021), responsible for Legionnaires’ disease, associated with high-risk pneumonia (Van der Kooij et al., 2017) has also been found. These microorganisms or genetic elements (such as ARGs, plasmids, and integrons) (Jin et al., 2020) can infiltrate the water system through compromised plumbing or cisterns exposed to contaminated water. Moreover, biofilms, which begin building up on submerged surfaces within the first week, can further facilitate their spread (Artiola et al., 2013).

### The contribution of high levels of NOM in water insecurity

In Canada, all public, semi-public, and private DWS are regulated by provincial and territorial authorities, with guidelines tailored to the specific type of source water used. (Province of Manitoba, 2007; Province of Manitoba, 2020; Bhatnagar & Sillanpää, 2017; Environment and Climate Change Canada, 2017). In potable water sources in the province of Manitoba (where the 4th highest number of indigenous people live), for example, the high levels of dissolved organic carbon or DOC (a component of NOM) often exceed 20 mg/L. However, the typical DOC concentration of surface water quality in the prairies is on average 8–12 mg/L (Sadrnourmohamadi, Goss & Gorczyca, 2013). NOM is generally contained in natural aquatic sources because of the interaction between the disruption of plants habiting in the geosphere and the by-product of bacteria, and eukaryotes (*i.e.,* algae) (Bhatnagar & Sillanpää, 2017). Seasonal changes, runoff of DOC from the land to the source of water (*i.e.,* in storms) can also influence the levels of NOM in aquatic environments (Findlay & Sinsabaugh, 2002; Saraceno et al., 2009; Winter et al., 2018). The presence of NOM in drinking water treatment has been associated with bacterial regrowth and reduced effectiveness in inactivating other microorganisms, such as bacteriophages, (Templeton, Andrews & Hofmann, 2005; Pham, Mintz & Nguyen, 2009; Jacquin et al., 2020) *Cryptosporidium* (Dai & Hozalski, 2002; DiCesare, Hargreaves & Jellison, 2012) among others.

Organic and inorganic complexes represent a source of energy for either heterotrophic or chemoautotrophic bacteria, respectively (Van der Kooij, 1992). This event allows transportation of both: the hydrophobic organic compounds present in NOM (Bhatnagar & Sillanpää, 2017; Winter et al., 2018; Wu et al., 2019; Dixit et al., 2021) and toxic heavy metals such as copper (Cu), arsenic (As), lead (Pb), mercury (Hg), cobalt (Co), iron (Fe) and chromium (Cr) (Liu et al., 2018; Rilstone et al., 2021). When NOM interacts with chlorine used for water disinfection, different halogenations and oxidations result in the formation of DBP which contain potential organic genotoxic compounds(McKie et al., 2015; Wu et al., 2019). For instance, trihalomethanes (THMs), and haloacetic acids (HAAs) are the DBP regulated in the Guidelines for Canadian Drinking Water Quality. The maximum acceptable concentration within Canada for these compounds is 100 µg/L for THMs (Health Canada, 2013a) and 80 ug/L for HAAs (Health Canada, 2010). However, it has been documented that more than 300 water systems in Canada with populations of less than 5,000 people have exceeded the maximum concentration of HAAs permitted (Table 1).

**Table 1:** Number of small drinking water systems (DWS) that exceeded haloacetic acid (HAA5) levels in Canada. Drinking water systems (DWS) with <5,000 users that exceeded 80 ug/L, the level of haloacetic acids (HAA5) permitted in Canada. In Newfoundland, for example, 50.91% of small DWS exceeded the level of HAAs established.

**Province**	**No. of DWS of <5,000 users per province**	**No. of DWS that exceeded limit of HAA5**	**Percentage of DWS that exceeded HAA5 in that region (%)**
Ontario	32	1	3.13 (%)
Quebec	27	11	40.74 (%)
Nova Scotia	38	16	42.11 (%)
Newfoundland and Labrador	220	112	50.91 (%)

Moreover, the additional costs associated with high levels of NOM are generally because an augmentation of the DOC produces an increase in the coagulation and filtration processes that drinking water treatments execute (Eikebrokk, Vogt & Liltved, 2004; Soros et al., 2019a). This represents a significant demand in coagulants such as aluminum sulfate (Al_2_(SO_4_)_3_), polymerized ferrous sulfate (PFS), poly-aluminum chloride (PAC) or chitosan to limit biological growth, and chemicals to adjust the pH of water (Eikebrokk, Vogt & Liltved, 2004; Yang et al., 2016; Soros et al., 2019).

### Urban drinking water methods and their limitations in application to Indigenous reserves in Canada

In Canadian urban settings such as Toronto, Winnipeg, Edmonton, and Regina, the drinking water processes to remove NOM and other water contaminants may include the following phases:

• Coagulation/flocculation: to aggregate and grow the size of the particles present and NOM.
• Sedimentation: to remove the suspended solids from water.
• Ozonation: to decompose NOM into low molecular weight fractions, and chemically destroy microbial cells (Zouboulis et al., 2007; Hammes et al., 2008; EPCOR, 2021; City of Winnipeg, 2021; Government of Ontario, 2021).
• Rapid sand filtration: commonly used to remove biodegradable organic matter, ammonium, and other organic micropollutants.
• Addition of chlorine: to inactivate remaining pathogenic and non-pathogenic microorganisms present
• UV disinfection: to induce biochemical inactivation of waterborne parasites(Li et al., 2019; EPCOR, 2021; City of Winnipeg, 2021).

As mentioned above, coagulation is the most commonly used method for removing NOM in Canada (EPCOR, 2021; City of Winnipeg, 2021; Government of Ontario, 2021). Coagulation reduces the repulsion forces and aims to transform dissolved organic matter into neutral particles by adsorption onto aluminum or iron-based coagulants (Edzwald, 1993). The particles efficiently accumulate through flocculation to be removed afterward by clarification (Edzwald, 1993; International Water Association, 2024). The downsides of applying coagulation in remote DWS include high costs and high doses of anticoagulants and other chemicals needed for pH modification (Eikebrokk, Vogt & Liltved, 2004; Soros et al., 2019). Depending on the water source and the specific conditions of the water to be treated, additional drinking water methods available include:

• **High-pressure membranes:** High-pressure membranes are advanced filtration methods developed to remove dissolved matter from water with a high energy demand. They are generally used in groundwater to reduce salt content, nitrates, and other organic and inorganic micropollutants (Besile, 2015; Frenkel, 2015). Some examples of high-pressure membrane filtration include reverse osmosis (RO) and nanofiltration (NF). RO is a pressure-driven technology that uses a semi-permeable membrane typically made of cellulose and polyamide. This enables the passing of water size particles but blocking all the solids, dissolved matters, colloids, salts, and organic matters with a molecular weight greater than 50 to 100 Da (Zhao, 2018; Prasad, 2020). Likewise, NF uses mostly polymer membranes with larger pore sizes (100-1 nm) than the ones used in RO (Nguyen, Roddick & Harris, 2010; Zhao, 2018). The pressure used in NF is lower and thus consumes less energy than RO (Zhao, 2018). The limitations of RO and NF are the high cost of the membrane, maintenance, and biofouling(Follet & Hatfield, 2001; Nguyen, Roddick & Harris, 2010; Zhao, 2018). This method is not easy to implement in Indigenous reserves due to high energy demand (which is commonly not available in remote communities), costs associated with membrane replacement, and highly trained operator requirements (Follet & Hatfield, 2001; Zimmermann et al., 2020).
• **Low-pressure membranes:** Low-pressure membranes are advanced methods for water treatment to remove macromolecules (such as dyes, proteins, polysaccharides) with low energy demand. Generally, these membranes are not capable of eliminating all dissolved organic matter alone because of membrane fouling. Therefore, an appropriate water pre-treatment is required (Davoodbeygi et al., 2023). Some examples of low-pressure membrane processes include microfiltration (MF) and ultrafiltration (UF). These membranes are commonly used after the employment of coagulants. This improves the permeate flux and helps to avoid membrane fouling (Nguyen, Roddick & Harris, 2010). Notably, MF and UF are effective for particle and microbial removal, the drawback is that the membranes used for these methods are recognized to be fragile and costly (Hoslett et al., 2018). Additionally, in most cases, only a fragment of the organic components is removed (Hoslett et al., 2018). MF and UF membrane treatments fail to remove significant levels of NOM (Jacangelo, Trussell & Watson, 1997; Hoslett et al., 2018). Consequently, low-pressure membrane filtration systems are contemplated as pre-treatments of other sophisticated methods such as NF or RO and the cost outweighs their implementation in the DWS of Indigenous communities (Hoslett et al., 2018).

• **Ozone:** Ozone is an advanced oxidation process that is useful for breaking organic chains and detaching aromatic rings in recalcitrant organic complexes along with efficient microorganism inactivation (Zouboulis et al., 2007; Loh et al., 2021).
• **Biological activated carbon (BAC).** BAC filters are a type of water treatment that combine the adsorption of activated carbon with the biodegradation of microorganisms to purify water. In this type of filter, activated carbon is used as a carrier that supports the growth of microorganisms, which subsequently degrade organic compounds (Jin et al., 2013). Ozone is often paired with BAC filtration. BAC filters following ozonation have shown significant effectiveness in removing NOM, ozonation by-products, DBP precursors, as well as taste and odor compounds (Simpson, 2008; Hamid et al., 2019). There are more than 800 water facilities using ozone as part of their water purification system across Canada (Health Canada, 2016). Some disadvantages of ozone treatment, depending on the water matrix (surface water or wastewater), and/or residual pollutants, are the incomplete degradation of organic substances which results in the generation of pathogenic by-products (*i.e.,* brominated organics, aldehydes, and carboxylic compounds) (Huang, Cheng & Cheng, 2007). Furthermore, as a result of ozonation, DOC levels could increase because of the liberation of extracellular organic matter and other proteins and polysaccharides (Huang, Cheng & Cheng, 2007). Moreover, high ozone doses are required for the successful inactivation of microorganisms (Zouboulis et al., 2007; Manasfi, 2021). Lastly, the lifetime of ozone is brief and needs on-site production which is not an option for water systems on Indigenous reserves due to their remote locations (Zimmermann et al., 2020). Regarding the limitations of BAC systems, their gradually decreasing absorption capacity over time and low DOC removal should be noted (Zimmermann et al., 2020). When dissolved organic matter and other complex compounds are not adequately removed during the water treatment, pathogenic microorganisms can proliferate inside the water distribution system (Okabe, Kokazi & Watanabe, 2002; Simpson, 2008; Hamid et al., 2019). Without proper maintenance, this method represents a health risk for the consumers in addition to poor water quality (Okabe, Kokazi & Watanabe, 2002).
• **Ion exchange** **(IEX).** IEX is an electrochemical method where ions from the water body are swapped with ions within resins that contain active centers with acidic or basic groups (electrically charged) (Gefter, 1962; Keller, 2005; Drioli, Giorno & Fontananova, 2017; Al-Asheh & Aidan, 2021). The most common materials used for IEX resins include methacrylic acid, sulfonated styrene, and divinyl benzene (DVB) (Brady et al., 2017). Cation exchangers carry sulfate or carboxyl groups and use sodium, potassium, and hydrogen as counterions (Brady et al., 2017). Whereas anionic ion exchange resins contain quaternary ammonium groups with chlorine as a counterion (Brady et al., 2017). During water treatment for DOC removal, anionic IEX takes place when the negatively charged dissolved organic matter in waters with pH values ranging from 6 to 8 (which is the case of most drinking water sources) has a higher attraction for the ion exchange resin than the ion being traded (Arias-Paic et al., 2016). The removal of anions releases a Cl − ion from the anionic resin (Arias-Paic et al., 2016). Anionic IEX can eliminate up to 90 percent of DOC (Dixit, Barbeau & Mohseni, 2020; Zimmermann et al., 2020). Additionally, the cost of a traditional IEX system fluctuates around $0.1–$0.2 per 1,000 liters of purified water, which is significantly lower than other membrane-based methods (Dixit, Barbeau & Mohseni, 2020). Removing DOC from water through IEX may be considered a viable, efficient, and affordable alternative for rural systems as it does not require continuous operational capacity or personnel to operate other treatment processes. The drawbacks of IEX implementations include the saturations of resins with chloride counter-ions after 3 to 8 weeks of performance (depending on DOC levels, conditions of operation, and IEX capacity), which requires frequent regeneration (Amini et al., 2018; Winter et al., 2018; Zimmermann et al., 2020). The regenerations produce elevated concentrations of sodium chloride and NOM that demand careful disposal since it can negatively affect the aquatic ecosystem and the plumbing structure of the area if disposed directly into the sewer sheds (Rokicki & Boyer, 2011; Liu et al., 2020). Thus, the constant transportation of regenerants (10–12% NaCl solution) represents a clear disadvantage for remote Indigenous Communities (Rokicki & Boyer, 2011; Liu et al., 2020). In IEX, the favorable conditions of the macro-porous anatomy of the resin and the presence of high sources of carbon, benefit biofilm development in the membrane in the absence of resin regeneration (Flemming, 1987; Schulz et al., 2017). The establishment of biofilm was considered undesirable, nevertheless, several recent studies have demonstrated that biological activity found in the IEX resins contribute to the removal of NOM (Schulz et al., 2017; Amini et al., 2018; Winter et al., 2018; Liu et al., 2020; Zimmermann et al., 2021). This high-performance method, now called Biological Ion Exchange (BIEX) enables the saturated membrane of IEX to extend its lifetime without filter maintenance, minimal waste discharge, and low operational cost (Amini et al., 2018; Liu et al., 2020; Zimmermann et al., 2021). BIEX has recently been applied in the drinking water system used by the Middle River community located in central British Columbia. This Indigenous reserve, which is part of the Tl’azt’en Nation, has been under drinking water advisory for over a decade (Zimmermann et al., 2020). The implementation of BIEX in this community resulted in high DOC removal, with no need for replacement of the filter in more than 12 months (Zimmermann et al., 2020). Remarkably, after the implementation of BIEX in this community, the drinking water advisory was lifted (Zimmermann et al., 2020). Although there is not yet a consensus reached regarding the percentage of DOC removed by the biological activity of the resins used in this method, strong evidence suggests that biodegradation may significantly influence DOC levels (Zimmermann et al., 2021). This method can be applied in complementation with UV disinfection, an advanced oxidation process, and chlorine for NOM elimination (Zimmermann et al., 2020). Further research is, however, required to elucidate the dynamics of biofiltration in NOM removal through IEX. It is critical to identify accessible technologies capable of removing NOM and treating source water in DWS of Indigenous communities, particularly for those under long-term drinking water advisories. This will ensure compliance with water quality standards and safeguard the health of all users.

### Drinking water treatments in Indigenous communities of other high-income countries

Although information on DWS in Indigenous communities worldwide is limited, reports indicate that many of these communities’ face challenges similar to those in Canada. Using untreated groundwater, storage, and transportation facilities such as cisterns and water trucks seems to be a common denominator. In the USA, reports revealed that approximately 12% of Native Americans including Alaska Native communities do not have basic sanitation facilities (White, Murphy & Spence, 2012). In the Navajo Nation, one of the largest Indian reservations in this country, studies evidenced that the DWS had a very basic infrastructure consisting of wells, pressure, and distribution tanks operated by the owners. In this Nation, around 30% lacked piped water (VanDerslice, 2011; Ed, 2024). A high number of “tribal facilities” (Conroy-Ben & Richard, 2018) exceeded the safe limits established by the US EPA drinking water regulations for fecal bacteria and heavy metals including arsenic and uranium (VanDerslice, 2011; Ed, 2024). In Australia, most Indigenous people rely on untreated groundwater that has not met the recommended limits for chemical and microbiological parameters by the Australian drinking water guidelines (Anonymous, 2011; Rajapakse et al., 2019; Balasooriya, Rajapakse & Gallage, 2023). In Greenland (Denmark), where more than 80% of the population is Indigenous (Cardenas, 2022), source water is treated with sand filters, UV and/or chlorination. To transport water to houses without piped water (approximately 10%) water trucks bring water inside households’ storage units. Another alternative is the existence of tap houses where people fetch water (Gunnarsdottir et al., 2020). Although records are limited or absent in small DWS in Greenland, some reports have detailed water facilities not meeting the microbiological European Union drinking water regulations (EU DWR), and BWA are frequent (Gunnarsdottir et al., 2020). Within the same context, Indigenous populations such as the Sámi people who are mostly distributed (∼95%) in Northern parts of Norway, Sweden, and Finland face challenges related to water and land (Nordic Arctic cooperation, 2013; Rauna, 2023; Stockholm International Water Institute, 2019). Although specific reports on the DWS infrastructure used in the communities of the Sámi people are not available, most small DWS in these Nordic countries use groundwater as their main water source (Gunnarsdottir et al., 2020). In Finland and Norway for example, small DWS typically have dug wells or boreholes. Generally, water from these DWS does not go under any treatment before distribution unless the source water has been demonstrated not to be “well protected” (Pitkänen et al., 2011). While the water quality has been cataloged to meet the minimum standards of the EU DWR, there are reports indicating water fecal contamination with *E. coli* (which doesn’t overrule the presence of other pathogens) in DWS of both countries (Pitkänen et al., 2011; Gunnarsdottir et al., 2020). In small DWS of Sweden, untreated groundwater is also distributed unless high levels of iron and manganese are found. If water treatment is required, water is aerated, filtered with rapid sand filtration, and disinfected. Sometimes, groundwater also goes through activated carbon filters (Gunnarsdottir et al., 2020). The monitoring of water supplies has been reported to be either absent or insufficient in Greenland (Denmark) and Sweden, respectively. The data reveal the vulnerability and ineffectiveness of the DWS that Indigenous communities face around the world.

### Importance of determining the microbiome inhabiting DWS

One of the main goals of drinking water treatments in Canada is to remove NOM since its presence is problematic because of the potential formation of DBP (McKie et al., 2015; Wu et al., 2019), contribution to biofilm growth (Templeton, Andrews & Hofmann, 2005; Pham, Mintz & Nguyen, 2009; Jacquin et al., 2020), and high operational costs (Eikebrokk, Vogt & Liltved, 2004; Yang et al., 2016; Soros et al., 2019) as previously reviewed. The use of biological-activated methods in drinking water treatment plants has proven to have a positive impact on the degradation of a fraction of NOM (Zhang et al., 2017). Even though some micropollutants need specialized electrochemical degradation (Asadi Zeidabadi et al., 2023), the effective depletion of contaminants including pharmaceuticals, personal care products, endocrine disrupting compounds, arsenic, manganese, and ammonium, has been well reported (Zhang et al., 2017; Wagner et al., 2018; Kirisits, Emelko & Pinto, 2019; United States Environmental Protection Agency, 2020). In this context, biofilters assist in the reduction of membrane fouling, color and odor constituents, disinfectant doses, and DBP precursors (Cuthbertson et al., 2019; Kirisits, Emelko & Pinto, 2019; Verdugo et al., 2020). A better understanding of the physiological, metabolic, biochemical, and ecological characteristics of the microbial networks found in DWS can be achieved by the identification of their genome (Willey, Sherwood & Woolverton, 2011; Bruno et al., 2018). Understanding the growth conditions, symbiotic relationships, pathogenicity risks, and habitat preferences of the microorganisms present in DWS and raw water is critical for making water safe. The aforementioned factors also provide insights to alter the conditions surrounding the microbiota and modulate the development of biofilms (Bruno et al., 2018). Regulating the microbial communities in biologically activated filters used in DWS could lead to the development of advanced approaches that positively impact various aspects of water safety. For instance, monitoring the microbiome in both water filters and source water could facilitate the effective prevention of high-risk pathogens (Bruno et al., 2018). In addition, when using BAF, efforts should be made to provide an ideal working environment for the microorganisms contributing to the degradation of water contaminants (Kirisits, Emelko & Pinto, 2019). These efforts will allow the recognition of the correlation between microbial community structure and biofilter function (Kirisits, Emelko & Pinto, 2019). The customization of the microbial community present in BAF according to the definitions proposed by Kirisits, Emelko & Pinto (2019) includes 3 approaches:

• Bioaugmentation: with the inoculation of pivotal endogenous or exogenous microorganisms from an enriched source to the one of interest to accelerate the normal microbial establishment process and fasten biodegradation (Lauderdale et al., 2012; Kirisits, Emelko & Pinto, 2019).
• Amendment: that refers to the adjustment of exogenous compounds required for biological activity such as nutrients and oxidants (Lauderdale et al., 2012; Kirisits, Emelko & Pinto, 2019); and
• Supplementation: of endogenous substances to achieve higher amounts than the ones contained naturally within the filters (Kirisits, Emelko & Pinto, 2019).

Although not considered in most operative water systems using BAF, the enhancement of biological activities has demonstrated a significant improvement in different aspects of the water filtration process (Albers et al., 2018). For example, in a bioaugmentation experiment conducted by Albers et al. (2018), enriched nitrifying bacteria from an operational sand filter was transferred to a novel filter, resulting in an acceleration of the development of key nitrifiers in the filters (Pinto et al., 2016; Albers et al., 2018; Kirisits, Emelko & Pinto, 2019). This approach enables speeding the oxidation of ammonium in groundwater, a process that under normal conditions would take several months (Pinto et al., 2016; Albers et al., 2018; Kirisits, Emelko & Pinto, 2019). In another study conducted by Lauderdale et al. (2012), the amendment of nutrients and peroxide resulted in filter life extension as well as a breakthrough decrease of DOC and other undesirable components (Lauderdale et al., 2012). In contrast, these improvements were not observed with other BAF systems used without this enhancement (Lauderdale et al., 2012). Moreover, in research conducted by Keithley & Kirisits (2019), it was proposed that supplementation of phosphorus in the biofilters only when P levels are limited helps to ameliorate biofilter hydraulics (Keithley & Kirisits, 2019). In natural conditions, phosphorus is low, due to removal through conventional treatments (*i.e.,* coagulation/flocculation) (Keithley & Kirisits, 2019). The deficit of P contributes to an increase in the extracellular polymeric substances of the biofilm which has been correlated with bio-clogging and less filter durability (Xia et al., 2014; Keithley & Kirisits, 2019). Therefore, more efforts should be considered to determine the microbiome established in the biofilters to increase their durability and secure correct functioning in DWS.

Identifying the microbiome present in biological water filters represents the cornerstone for finding putative functions associated with these microorganisms. Thereby, any attempt of amendment, supplementation, and/or bioaugmentation to improve the biodegradation of any organic or inorganic component, should be complemented with the microbial fingerprints present in the biofilter to link activity and symbiosis of the microbiome (Lauderdale et al., 2012; Kirisits, Emelko & Pinto, 2019).

### Metagenomics

Culture-dependent methods have been widely used as the technique of reference for controlling the presence of pathogenic organisms in water. However, these methods do not provide significant information regarding the total microbial diversity and its changes in source water and DWS (Douterelo et al., 2014; Uyaguari et al., 2016). Moreover, some commercial kits used for fecal bacteria quantification in water (*i.e.,* compartment bag tests) have been demonstrated to underestimate the concentration of pathogenic bacteria. Wang et al. (2017), which increases the risks of false negative results. Besides, most bacterial cells present in the water as well as in BAF and DWS are not culturable or are out of the range of detection of commercial tests (Theron & Cloete, 2000; Bruno et al., 2018; Lewis et al., 2021). To obtain information about the type, abundance, function, pathogenicity, and metabolic requirements of the drinking water microbiome, culture-independent or molecular methods are the approach of choice (Theron & Cloete, 2000; Boughner & Singh, 2016). These high-throughput methods can provide more detailed information to monitor BAF, enabling targeted modifications that can enhance the performance of these filters in DWS (Theron & Cloete, 2000; Boughner & Singh, 2016; Kirisits, Emelko & Pinto, 2019). Moreover, the rapid decline in the cost of these sequencing tools makes them conveniently affordable, even for DWS in Indigenous reserves (Hull et al., 2019). Up to date, Metagenomics, which involves studying all nucleic acids from a specific sample, has become the preferred method for microbiome analysis, especially using targeted or amplicon-based metagenomic approaches (Eils & Kriete, 2014; Kibegwa et al., 2020). One of the main reasons for these preferences is the high level of conservation and hyper-variability of the genomic marker used, which allows for the identification of different species (Douterelo et al., 2014; Uyaguari et al., 2016; Kibegwa et al., 2020). The multiple sets of DNA sequences identified during high-throughput sequencing can be used to evaluate the taxonomy of the microorganisms present in drinking water and biofilter microbial communities (Kirisits, Emelko & Pinto, 2019). For instance, deep amplicon sequencing of 16S rRNA and chaperonin 60 or cpn60 (mitochondrial protein) has been used for identifying bacterial communities in source water and DWS (Baron et al., 2014; Uyaguari et al., 2016; Brumfield et al., 2020). Other microorganisms such as eukaryotes and fungi have also been described in aquatic environments using 18S rRNA and Internal Transcribed Spacer (ITS) (Weber et al., 2009; Pereira et al., 2010; Schoch et al., 2012; Uyaguari et al., 2016). Similarly, characterization of the viruses found in drinking water has been attempted by studying specific viral groups using biomarkers such as RNA-dependent RNA polymerase (RdRp) for RNA virus, gene 23 (g23), and gene 20 (g20) for DNA virus, among others (Zheng et al., 2013; Uyaguari et al., 2016; Wang et al., 2016). Despite the virome characterization obtained using the mentioned viral biomarkers, approximately 50% of viral hits remain unknown when searched in public databases (Barrientos-Somarribas et al., 2018). The hassle relies on viruses lacking “universal” gene markers which makes the identification of abundance patterns and community structure for all viruses a challenge (Brum et al., 2015; Kulski, 2016; Uyaguari et al., 2016). Despite this, several viruses have been associated with human fecal contamination such as Norovirus, Enterovirus, Rotavirus, Hepatovirus A, Pepper mild mottle virus (PMMoV), crAssphage, and human adenovirus (HAdV), among others (Wong et al., 2012; Holcomb & Stewart, 2020). Additionally, depending on the source of water, animal-specific enteric viruses can also serve as fecal indicators, such as the case of porcine and bovine adenoviruses’ ((PAdVs) and (BAdVs), respectively) as well as bovine enterovirus (BEV) and Bovine polyomavirus (BPyV) (Xagoraraki, Yin & Svambayev, 2014). Table 2. summarizes the different viruses and other microorganisms used to assess fecal contamination in aquatic environments. The prevalence of these microorganisms has been identified in multiple water environments (McMinn, Ashbolt & Korajkic, 2017), for this reason monitoring source water, DWS and biofilters with this technology can help ensure adequate water quality for all individuals.

**Table 2:** Microorganisms used to assess fecal contamination in water. Fecal indicator bacteria (FIB), fecal indicator viruses (FIV) and common waterborne parasites. Under the FIB category, the groups, or species with an asterisk (*) represent complementary alternatives to the FIB most commonly used (*E. coli*, enterococcus, and total coliforms). Below the FIV section: FRNA phage= specific ribonucleic acid; PMMoV= Pepper mild mottle virus (plant virus); TMV= Tobacco mosaic virus (plant virus); AdV*= Human (HAdV), Porcine (PAdVs) and bovine (BAdVs) adenoviruses; AstVs= Astroviruses; EV*=human (HEV) and bovine (BEV) enteroviruses; HepV= Hepatitis viruses; NoV= Noroviruses; PyV*= human (HPyVs) and bovine (BPyV) polymoviruses; RV= Rotaviruses. Finally, beneath waterborne parasites, the species with an asterisk (*) are the less commonly used as a reference in this category.

**Fecal indicator bacteria**	**Fecal indicator virus**	**Waterborne parasites**
*Escherichia coli*	Coliphages	*Giardia lamblia*
*Enterococcus* spp.	*Bacteroides* bacteriophages	*Cryptosporidium parvum*
Fecal *streptococci*	*Enterococci* phages	Toxoplasma gondi*
Total coliforms	crAssphage	Cyclospora cayetanensis*
Bacteroides-Prevotella group*	FRNA phage	Entamoeba histolytica*
Bifidobacterium spp.*	PMMoV	Blastocystis hominis*
Clostridium perfringens*	TMV	
	AdV*	
	AstVs	
	EV*	
	HepV	
	NoV	
	PyV*	
	RV	

In addition to targeted metagenomics, another culture-independent approach is shotgun metagenomics, in which the contents of the complete genome are studied (Kibegwa et al., 2020). In this approach, long DNA molecules belonging to the microorganisms present in the ecosystem under study, break into random fragments that are sequenced afterward (Durazzi et al., 2021). Shotgun sequencing examines all metagenomic DNA instead of only the hypervariable regions (Kibegwa et al., 2020). When the nucleotide detection of target DNA molecules is direct, the efficacy of data analysis increases, and the coverage regions of phylogenetical relevance are extended (Sato et al., 2019). The average prices of targeted and shotgun metagenomics can be found in Table 3. Even though these tools have the potential to provide important information regarding the microbial populations present in source water, DWS and biofilters, there is still evidence of a vast number of uncategorized, uncharacterized, and unclassified environmental microorganisms (Lynch & Neufeld, 2015; Bruno et al., 2018; Lagier et al., 2018). For this reason, complementary methods should be used to fill in the existing taxonomic “blind spots” that separate the field today from vital and novel phylogenetic information.

**Table 3:** Current prices of “Omic” technologies. Services for metagenomics are accessible through different public and private institutions around Canada and the world. The manufacturing cost of less common technologies for drinking water systems (DWS) such as culturomics and paper-based microfluidic are included.

**Technology**	**Commercial price range per 1 sample (CDN$)**
Targeted metagenomics	$20–$50
Shotgun metagenomics	$130–$340
	**Manufacturing price per 1 sample (CDN$)**
Culturomics^*^	$8.00–$10.00
Paper-based microfluidics ^**^	$0.30–$4.00

**Notes.**

* Requires initial inversion of hardware and devices around 400,000.

** Cost based on 10 million devices production.

### Culturomics

Culturomics is a high-throughput culture approach that can be used to overcome the limitations of unclassified environmental bacteria that metagenomics faces (Lagier et al., 2018; Nowrotek et al., 2019). This technique was originally introduced for the study of microbiota in the human gut (Lagier et al., 2016). This method combines matrix-assisted laser desorption/ionization-time of flight (MALDI–TOF) mass spectrometry with 16S rRNA sequencing to identify novel, viable bacterial colonies (Lagier et al., 2016; Lagier et al., 2018). Its principle of improving culture media with the precise conditions these microorganisms require for their growth can help fill in the gaps of the so-called “uncultivable” bacteria of aquatic environments. In culturomics, diverse adjustments are applied to the incubation conditions (temperature, incubation time, media enrichment, pH, oxygen demand, and so forth) to promote the growth of otherwise uncultivable bacteria (Nowrotek et al., 2019). Consecutively, they are cleaned and prepared for the MALDI-TOF method, and if the taxonomic credentials in the database are not found, a supplementary amplification and sequencing using 16S rRNA is conducted (Nowrotek et al., 2019). The transcendental results obtained from the human gut microbiome (247 new species of bacteria and their genomes unveiled) make this method a suitable candidate to complement metagenomics for the uncategorized phyla found in source water and DWS (Lagier et al., 2016). Conversely, the limitations of culturomics lie in the inability to identify species that do not count with any genome registration in libraries of reference (Lagier et al., 2016; Lagier et al., 2018). Furthermore, the sample-processing capacity is pointedly lower compared to the volume that metagenomics tools can handle in a single day (Lynch & Neufeld, 2015).

### Microfluidics or Lab-on-a-chip technologies

Indigenous and remote communities would significantly benefit from fast, transportable, and on-site sensitive methods to recognize bacterial, viral, and protozoan pathogens in DWS. The term microfluidics refers to the process of small (10^−9^ L to 10^−18^ L) fluids (Meissner, Seddon & Royall, 2019) that circulate into micrometer channels with components like microfilters, microvalves, micromixers, and sensors such as detectors at the cellular and molecular level (Guo et al., 2015; Tommaso, 2019). Generally, the channel size used in the analytical devices employed in microfluidics oscillates between 10 mm to 200 mm and even one mm in some cases (Guo et al., 2015). Lab-on-a-chip (LOC) employs a microsystem where the surface, gravitational, viscous, and other forces integrated with either active or passive microvalves are carefully applied to obtain a real and complete micro-laboratory (Guo et al., 2015; Tommaso, 2019). Active microvalves require external actuation (*i.e.,* electromagnetism, thermal expansion) while passive valves base their functioning on the pressure gradient (Pan et al., 2005; Guo et al., 2015). These (passive) microvalves are generally used for micropumps (*i.e.,* as check-valves) (Pan et al., 2005; Guo et al., 2015). Nichols et al. (2010) tested an insolation chip (ichip) composed of more than a thousand miniature diffusion chambers to inoculate microorganisms from diverse environments including aquatic settings (Nichols et al., 2010). The application of this technique has resulted in a higher recovery compared to traditional cultivation methods alone (Nichols et al., 2010). Additionally, the species found differed significantly from the ones recovered in Petri dishes, revealing considerable phylogenetic novelty (Nichols et al., 2010).

The specific functioning, types, and components of microfluidics have been broadly studied in different areas and can be revised elsewhere (Nichols et al., 2010; Guo et al., 2015). Generally, there are more than five types of microfluidic platforms including linear actuated devices, microfluidic large-scale integration, centrifugal microfluidics, segmental flow microfluidics, electrokinetics, surface acoustic waves, pressure-driven laminar flow, and lateral flow tests (Mark et al., 2010; Nichols et al., 2010). For instance, centrifugal microfluidics allows the management of more sensitive liquids such as nucleic acids (Mark et al., 2010). Some examples of the application of centrifugal microfluidics include DNA extraction and nucleic acid–based assays, protein crystallization and protein-based assays, integrated plasma separation, clinical chemistry assays, and chromatography tests (Mark et al., 2010).

Similarly, lateral flow tests, have been successfully used to detect infectious agents such as *Salmonella spp.*, anthrax (*Bacillus anthracis*), viruses, and even small molecules such as antibiotics (Ho et al., 2008; Mark et al., 2010). Some of the samples used in lateral flow tests include: nucleic acids for *B. anthracis* (Carter & Cary, 2007), blood serum for *Salmonella spp.* (Ho et al., 2008), nasopharyngeal wash for respiratory syncytial virus Infection (RSV) (Mokkapati et al., 2007), milk for tetracycline detection(Chen et al., 2019) and fecal specimens for *Giardia spp.* and *Cryptosporidium spp.* (Johnston et al., 2003; Rasooly et al., 2017). In lateral flow tests, the capillary-driven process is used to absorb the sample and transport it through all over the test strip. Three types of molecules or antibodies will be present in this mechanism where:

• Tagged antibodies for a signal-generating particle in the “conjugate pad” are hydrated with the sample and eventually bound to the antigens contained in the sample(Mark et al., 2010; Wei et al., 2023).
• The sample continues flowing to the incubation and recognition area and meets test line antibodies that bind particles covered with antigens (Mark et al., 2010).
• On the control line, a third class of antibodies catches the compounds that did not bind with any particle (Mark et al., 2010). This latter binding confirms a successful test performance (Mark et al., 2010). Likewise, the binding or not binding antibodies in the detection line confirm or deny the presence of the analyte of interest (Mark et al., 2010; Wei et al., 2023).

To the best of our knowledge and based on current literature, these methods have not yet been used to test water microbial quality. In this context, lateral flow tests embody a cost-effective, mobile, and top-notch alternative to identify well-known pathogens in the drinking water systems of Indigenous reserves.

The mechanism of action that microfluidics has, could be replicated for the detection of fecal indicator bacteria, viruses, and protozoans in the source water and DWS of isolated areas and Indigenous communities. Nevertheless, the drawbacks of microfluidics include system blockage by small elements, as well as high contamination risks with minimal amounts, and premature absorption of the analytes of interest, among others (Guo et al., 2015). Microfluidics platforms represent low-cost, portable, high-precision, time-optimizer tools that would benefit DWS with higher susceptibility to microbiological contamination as is the case of DWS located in remote Indigenous reserves (Guo et al., 2015; Reboud et al., 2019).

## Recommendations and Conclusion

This review highlights that water insecurity in Canada is a multicomplex event perpetuated by several factors. The first one started with the systematic exclusion of Indigenous peoples to geographically isolated reserves, which came with a lack of planning for policy frameworks and basic infrastructure of drinking water facilities. Moreover, the fragmented responsibilities between the federal, provincial, and territorial governments in Canada along with FN, in the design, construction, operation, and financing of DWS, contribute to this long-lasting issue. The second factor can be associated with the deterioration of water and the environment and the obsolete or non-existent DWS in Indigenous reserves, which are inadequate to address microbiological and chemical contaminants. In Canada some water advisories in Indigenous reserves have not been lifted for almost 30 years (Indigenous Services Canada, 2024a). In places where traditional drinking water treatments are not available due to underfunding or lack of support from authorities or source-water quality issues (including high NOM levels), efficient and long-term alternatives should be implemented to permanently lift drinking water advisories. To improve DWS under advisories, comprehensive design studies are required in addition to provisional and permanent repairs in their infrastructure. The most common methods to treat drinking water and to remove NOM are coagulation, high- and low-pressure membrane filtration procedures, ozone, IEX, and its variant BIEX. Every method has its advantages and limitations, however, the method to be implemented should be specific to the source water conditions of the area to be implemented. Furthermore, source water protection, biofilter management, and drinking water monitoring is fundamental to prevent the negative health effects of pathogenic microorganisms. Metagenomics and its drop in prices represent an effective tool to monitor the water microbiome. Additionally, technologies such as culturomics can exceptionally contribute to revealing the up-to-date “unclassified” microbial diversity in DWS. Once identifying the microbial fingerprint in source water and DWS, practical approaches such as lab-on-chip technologies can be implemented for on-site, ultra-fast water microbial quality assessment. Simplifying water quality monitoring will significantly enhance the promotion of health and access to safe water, principally for consumers in remote Indigenous communities.

Providing culturally adapted water services must be a priority to ensure appropriate water quality and availability. For that, a dynamic interplay between FN authorities and federal, provincial, and territorial governments is imperative to help prioritize and assign resources to promote water protection and management in Indigenous reserves of Turtle Island.

## Nomenclature

BAF: Biological activated filters
DBP: Disinfection by-products
DWS: Drinking water systems
DOC: Dissolved organic carbon
FN: First Nations
NOM: Natural organic matter

## Additional Information and Declarations

### Data Availability

The following information was supplied regarding data availability: This is a literature review.
